# After all, plasmalemmal expression of type-1 VDAC can be understood. Phosphorylation, nitrosylation, and channel modulators work together in vertebrate cell volume regulation and either apoptotic pathway

**DOI:** 10.3389/fphys.2015.00126

**Published:** 2015-04-27

**Authors:** Friedrich P. Thinnes

**Affiliations:** Max-Planck-Gesellschaft MünchenGöttingen, Germany

**Keywords:** porin, VRAC, VSOAC, ICl_swell_, cystic fibrosis, Alzheimer's Disease, malaria, autism

## Introduction

Since the beginning of VDAC's history in 1976 artificial lipid bilayer studies of native (Schein et al., 1976; Benz et al., 1992; Colombini and Mannella, 2012) and recombinant (Bayrhuber et al., 2008; Hiller et al., 2008; Ujwal et al., 2008; Teijido et al., 2012) VDAC preparations are used to operationally define differing states of VDAC channel regulation. Up to near past just one fully open and a series of partially closed states have been stated. Since 1989, human type-1 VDAC was shown to reside in the plasmalemma of mammalian cells, a fully closed VDAC-1 state has been postulated, putatively achieved by interactions with proteineous modulators, low molecular weight agonists, or posttranslational protein modifications, as there are acetylation, phosphorylation, and nitrosylation (Thinnes and Reymann, 1997; Thinnes, 2013). Correspondingly, three differing VDAC states are discussed in recent structural studies on type-1 vertebrate VDAC using varying biophysical or *in silico* approaches (Bayrhuber et al., 2008; Hiller et al., 2008; Ujwal et al., 2008; Teijido et al., 2012). These are: (1) open = anion-selective = N-terminal stretch of AA 1-25 inside the barrel; (2) partially closed = cation-selective = N-terminal stretch of AA 1-25 inside the barrel but in differing position; (3) fully closed = collapsed = impermeable for ions or flickering = N-terminal stretch outside the β-barrel and accessible for antibodies.

## Phosphorylation of VDAC-1 of cell in culture helps understand conductance switches induced by amyloid Aβ

Fernandez-Echevarria et al. (2014) summarize their recent study linking regulation of plasmalemma-standing VDAC-1 and the etiology of Alzheimer's Disease as follows:

“Voltage-dependent anion channel (VDAC) is a mitochondrial protein abundantly found in neuronal lipid rafts. In these membrane domains, VDAC is associated with a complex of signaling proteins that trigger neuroprotective responses. Loss of lipid raft integrity may result in disruption of multicomplex association and alteration of signaling responses that may ultimately promote VDAC activation. Some data have demonstrated that VDAC at the neuronal membrane may be involved in the mechanisms of amyloid beta (Aβ)-induced neurotoxicity, through yet unknown mechanisms. Aβ is generated from amyloid precursor protein (APP), and is released to the extracellular space where it may undergo self-aggregation. Aβ aggregate deposition in the form of senile plaques may lead to Alzheimer's disease (AD) neuropathology, although other pathological hallmarks (such as hyper-phosphorylated Tau deposition) also participate in this neurodegenerative process. The present study demonstrates that VDAC1 associates with APP and Aβ in lipid rafts of neurons. Interaction of VDAC1 with APP was observed in lipid rafts from the frontal and entorhinal cortex of human brains affected by AD at early stages (I-IV/0-B of Braak and Braak). Furthermore, Aβ exposure enhanced the dephosphorylation of VDAC1 that correlated with cell death. Both effects were reverted in the presence of tyrosine phosphatase inhibitors. VDAC1 dephosphorylation was corroborated in lipid rafts of AD brains. These results demonstrate that Aβ is involved in alterations of the phosphorylation state of VDAC in neuronal lipid rafts. Modulation of this channel may contribute to the development and progression of AD pathology.”

From my point of view this study marks a break through to understand the incorporation of VDAC-1 into cell membranes. For a recent review on VDAC phosphorylation concerning the cell fate see (Martel et al., 2014).

## Nitrosation of VDAC-1 is critical for blocking the channel in fully closed or flickering state

Tewari et al. (2015) by a combination of black membrane measurements of recombinant VDAC-1 and *in silico* work quite recently elaborated the relevance of nitrosation for blocking VDAC-1 in its closed state. While the authors discuss their data only in the context of mitochondria-standing VDAC-1, I read them as supporting the plasmalemmal expression of VDAC-1.

They summarize their work by: “The VDAC is the main conduit for permeation of solutes (including nucleotides and metabolites) of up to 5 kDa across the mitochondrial outer membrane (MOM). Recent studies suggest that VDAC activity is regulated via post-translational modifications (PTMs). Yet the nature and effect of these modifications is not understood. Herein, single channel currents of wild-type, nitrosated, and phosphorylated VDAC are analyzed using a generalized continuous-time Markov chain Monte Carlo (MCMC) method. This developed method describes three distinct conducting states (open, half-open, and closed) of VDAC activity. Lipid bilayer experiments are also performed to record single VDAC activity under un-phosphorylated and phosphorylated conditions, and are analyzed using the developed stochastic search method. Experimental data show significant alteration in VDAC gating kinetics and conductance as a result of PTMs. The effect of PTMs on VDAC kinetics is captured in the parameters associated with the identified Markov model. Stationary distributions of the Markov model suggest that nitrosation of VDAC not only decreased its conductance but also significantly locked VDAC in a closed state. On the other hand, stationary distributions of the model associated with un-phosphorylated and phosphorylated VDAC suggest a reversal in channel conformation from relatively closed state to an open state. Model analyses of the nitrosated data suggest that faster reaction of nitric oxide with Cys-127 thiol group might be responsible for the biphasic effect of nitric oxide on basal VDAC conductance.”

## Interaction of human plasminogen kringle 5 and plasmalemmal VDAC-1 links the channel to the extrinsic apoptotic pathway

Li et al. (2014) impressively widened knowledge concerning the proteineous VDAC-1 modulator human plasminogen kringle 5 (K5) by stating that:

“Human plasminogen kringle 5 (K5) is known to display its potent anti-angiogenesis effect through inducing endothelial cell (EC) apoptosis, and the voltage-dependent anion channel 1 (VDAC1) has been identified as a receptor of K5. However, the exact role and underlying mechanisms of VDAC1 in K5-induced EC apoptosis remain elusive. In the current study, we showed that K5 increased the protein level of VDAC1, which initiated the mitochondrial apoptosis pathway of ECs. Our findings also showed that K5 inhibited the ubiquitin-dependent degradation of VDAC1 by promoting the phosphorylation of VDAC1, possibly at Ser-12 and Thr-107. The phosphorylated VDAC1 was attenuated by the AKT agonist, glycogen synthase kinase (GSK) 3β inhibitor, and siRNA, suggesting that K5 increased VDAC1 phosphorylation via the AKT-GSK3β pathway. Furthermore, K5 promoted cell surface translocation of VDAC1, and binding between K5 and VDAC1 was observed on the plasma membrane. HKI protein blocked the impact of K5 on the AKT-GSK3β pathway by competitively inhibiting the interaction of K5 and cell surface VDAC1. Moreover, K5-induced EC apoptosis was suppressed by VDAC1 antibody. These data show for the first time that K5-induced EC apoptosis is mediated by the positive feedback loop of “VDAC1-AKT-GSK3β-VDAC1,” which may provide new perspectives on the mechanisms of K5-induced apoptosis.”

Again, this study strongly points to plasmalemma-integrated type-1 VDAC and furthermore links the channel to the extrinsic apoptotic pathway.

## Conclusion and outlook

A series of recent studies, from my point of view, marks great moments of VDAC research.

**Figure 1:**
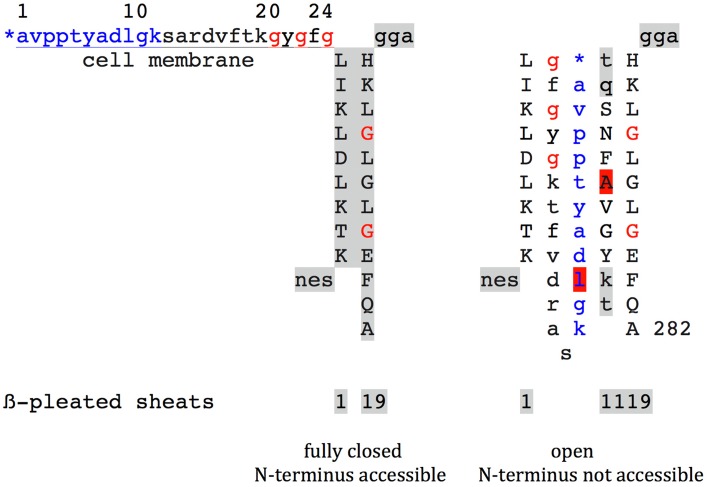
**Highly schematic two-dimensional projections of human type-1 VDAC summarising e data from my own lab and the laboratories of Vito De Pinto, Roland Benz, Varda Shoshan-Barmatz, Carmen Mannella, and Jeff Abramson for details see Thinnes, 2015**. Fully closed state and open state of native VDAC-1 are compared. Noteworthy the GxxxG motif, that has been shown to work as an ATP binding site and may putatively figure as a peptide interaction/membrane perturbation motif.

1. From my point of view, the recent studies of Fernandez-Echevarria et al. (2014), Tewari et al. (2015), and Li et al. (2014) are well in line with observations on cell membrane-expression, more precisely plasmalemmal lipid raft-integration of mammalian VDAC-1. The data on VDAC-1 posphorylation of cells in culture, this in correlation to channel opening and closing, help understand differing regulation states even in different compartments, and they also argue in favor of an involvement in cell volume regulation and thus apoptosis. The study on VDAC-1 nitrosation supplements those data by demonstrating its relevance for channel closing. The study of Li et al. (Best et al., 2007), furthermore, puts type-1 VDAC another time in touch with the extrinsic apoptotic pathway. Together, the results help to functionally understand much evidence indicating that plasmalemmal VDAC-1 forms the channel part of a volume regulated anion channel complex (VRAC, ORDIC, VSOR, Icl_swell_) that, in terms of molecular identity, still awaits definition (Dermietzel et al., 1994; Thinnes and Reymann, 1997; Thinnes et al., 2000; Okada et al., 2004; Elinder et al., 2005; Thinnes and Burckhardt, 2012; Thinnes, 2013; Akita and Okada, 2014).
2. There are increasing data pointing to the medical relevance of cell membrane-standing VDAC-1 by its involvement in the pathogenesis of several syndromes, e.g., cancer, cystic fibrosis, Alzheimer's Disease, autism, and malaria (Thinnes and Reymann, 1997; Best et al., 2007; Scharstuhl et al., 2009; Bouyer et al., 2011; Thinnes, 2013, 2014; Asmarinah et al., 2014; Shoshan-Barmatz et al., 2014; Tewari et al., 2014; Weiser et al., 2014; Zhang et al., 2014). These data, from my point of view, give reason for the request to keep plasmalemmal VDAC-1 on the schedule in mammalian VDAC studies.
3. Concerning the still running dispute on the accessibility of the N-terminus of VDAC at cell surfaces or outer mitochondrial membranes by antisera or monoclonal antibodies, respectively, Figure 1 (De Pinto et al., 1991; Guo and Mannella, 1992; Stanley et al., 1995; Geula et al., 2012; Tomasello et al., 2013; Huang et al., 2015; Thinnes, 2015). From my point of view, profit can be taken from an evident difference between animal and plant VDAC. While animal VDAC-1 at the proximal end of the free N-terminal peptide stretch carries a GxxxG motif, this motif is missing in plants (Thinnes, 2012). From here it may well pay to elaborate first three-dimensional structure date on plant VDAC, the more as new ideas to understand VDAC cell membrane incorporation may arise (Komarova et al., 2014).

### Conflict of interest statement

The author declares that the research was conducted in the absence of any commercial or financial relationships that could be construed as a potential conflict of interest.
